# Association Between Dietary Fiber Intake and Sleep Disorders: Based on the NHANES Database

**DOI:** 10.1002/brb3.70123

**Published:** 2024-11-05

**Authors:** Yijun Chen, Zengchen Zhao, Weijun Ding, Zhenting Zhou, Meihong Xiao

**Affiliations:** ^1^ Geriatric Medicine Department & Oncology Department Huzhou Nanxun Hospital of Traditional Chinese Medicine Huzhou City Zhejiang China; ^2^ Department of Rehabilitation Huzhou Traditional Chinese Medicine Hospital Affiliated to Zhejiang University of Traditional Chinese Medicine Huzhou City Zhejiang China; ^3^ Department of Neurology Huzhou Traditional Chinese Medicine Hospital Affiliated to Zhejiang University of Traditional Chinese Medicine Huzhou City Zhejiang China

**Keywords:** dietary fiber, logistic regression model, NHANES, sleep disorders

## Abstract

**Objective:**

Utilizing data from the National Health and Nutrition Examination Survey (NHANES) database, the primary objective of this investigation was to examine the relationship between dietary fiber intake (DFI) and sleep disorders.

**Methods:**

For analysis, data from three consecutive cycles of NHANES (2009–2014) were pooled. The independent variable of interest was DFI, while the dependent variable was sleep disorders. Weighted logistic regression was employed to model the relationship between the two variables. Subgroup analyses were conducted, stratified, and adjusted to explore the association between DFI and sleep disorders.

**Results:**

This study encompassed a cohort of 14,360 samples. Logistic regression results revealed a significant inverse association between higher DFI and the risk of sleep disorders (OR: 0.99, 95% CI: 0.98–1.00, *p* = 0.005). Stratified analysis demonstrated significant interactive effects of gender and physical activity on the association between DFI and sleep disorders (interaction *p* = 0.017, *p* = 0.061). Quartile‐stratified analysis of DFI showed that in the crude model, Q4 exhibited a significant protective impact against sleep disorders (OR: 0.76, 95% CI: 0.59–0.97, *p* = 0.026). In model I, which adjusted for demographic characteristics only, Q3 (OR: 0.74, 95% CI: 0.56–0.98, *p* = 0.036) and Q4 (OR: 0.70, 95% CI: 0.55‐0.90, *p* = 0.006) had significant protective effects on sleep disorders. Additionally, gender subgroup analysis revealed that DFI had a significant impact on the female population, particularly in postmenopausal women, and was more pronounced in subjects with BMI > 30 kg/m^2^ (*p* = 0.011). Within the physical activity subgroup, there was a certain effect of DFI on improving sleep disorders in individuals with low activity intensity.

**Conclusion:**

Increasing DFI had a protective effect in reducing the risk of sleep disorders. This protective effect may be more pronounced in the female population and individuals with low physical intensity.

## Graphical abstract

This study utilizes data from the National Health and Nutrition Examination Survey (NHANES) conducted between 2009 and 2014 to explore the association between dietary fiber intake and sleep disorders. The independent variable is dietary fiber intake, while the dependent variable is sleep disorders. Logistic regression analysis was employed to construct a relationship model between dietary fiber intake and sleep disorders, and stratified analysis was conducted on covariates to assess their impact on the model. The results indicate that increased dietary fiber intake is significantly negatively correlated with the risk of sleep disorders. Among women, particularly in the postmenopausal group with a BMI greater than 30 kg/m^2^, the effect of dietary fiber intake on improving sleep disorders is more pronounced. This study provides new dietary intervention ideas for improving sleep quality.

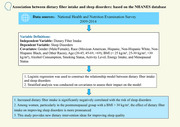

## 1 Introduction

Sleep disorders are a common disease that refers to the disturbance of an individual's normal sleep pattern, leading to difficulties falling asleep, decreased sleep quality, insufficient or non‐restorative sleep, and serious consequences for the individual's health and quality of life (Pavlova and Latreille 2019), as well as cognitive impairment (Chellappa and Aeschbach 2022), hypertension (Han et al. 2020), type 2 diabetes (Antza et al. 2021), metabolic syndrome (Simon et al. 2020), and cancer (Al Lihabi 2023; McDermott, Brown, and Chervin 2018; Buttner‐Teleaga et al. 2021), among other conditions. Common sleep disorders include insomnia, sleep apnea, and hypersomnia. Current treatment options include behavioral interventions, lifestyle modifications, medication, and device therapy, with dietary improvement being a common lifestyle modification (Perez and Salas 2020). In 2017, Meng et al. (2017) reported that grains, mushrooms, and sprouted beans in the diet can improve sleep efficiency and alleviate insomnia symptoms.

Dietary fiber, as an important nutrient in plant‐based foods, is abundant in grains, whole‐grain foods, nuts, and the outer layers and peels of vegetables and fruits. While indigestible by the human body, it exerts its influence by modulating the gut microbiota, contributing to digestive and overall bodily health (Guan, Yu, and Feng 2021; Swann et al. 2020), preventing intestinal problems such as constipation, hemorrhoids, and colon cancer (Song, Chan, and Sun 2020; Vernia et al. 2021), controlling blood sugar levels (Basu et al. 2021), lowering cholesterol (Schoeneck and Iggman 2021), controlling weight (Offringa et al. 2021), and promoting cardiovascular health. Recently, researchers have studied the linkage between dietary fiber and sleep disorders. In 2021, Hepsomali and Groeger (2021) did research on the linkage between diet and sleep‐related psychological health using the UK Biobank and found that high intake of vegetables, fruits, fish, water, and fiber was positively correlated with sleep and psychological health. In 2020, Gangwisch et al. (2020) conducted a feasible cohort study of postmenopausal women in the United States and found that high glycemic load is positively associated with the incidence of insomnia, while high dietary fiber intake (DFI) and whole grains are negatively linked with the incidence of insomnia. In 2023, Zhang et al. (2023) did a clinical cross‐sectional study and found a positive correlation between DFI and sleep quality in hemodialysis patients. Although the above studies have preliminarily identified associations between dietary fiber and various sleep‐related factors in different subgroups of populations, there is currently no comprehensive study on the linkage between DFI and sleep disorders among the general adult population. Hence, further investigation is warranted to better understand the relationship between dietary fiber and sleep disorders.

Here, we used the NHANES database and a weighted logistic regression model to re‐evaluate the association between DFI and sleep disorders and examined the impact of DFI on sleep disorders among various factors including age, race, gender, BMI, physical activity, smoking, and alcohol consumption.

## 2 Methods

### 2.1 Data Source and Study Population

The National Health and Nutrition Examination Survey (NHANES) database, a stratified, multistage study governed by the National Center for Health Statistics (NCHS) in the United States (http://www.cdc.gov/nchs/nhanes.htm), was used here. The database, freely accessible, serves the purpose of assessing the health and nutritional status of the U.S. population. It accomplished this by gathering data through interviews and physical examinations. The interview content encompassed demographic details, socio‐economic factors, dietary habits, and health‐related issues. The physical examination involved physiological measurements and laboratory tests. NHANES data were approved by the NCHS ethics review board and obtained with informed consent from participants and can be accessed at https://wwwn.cdc.gov/nchs/nhanes/Default.aspx.

**FIGURE 1:**
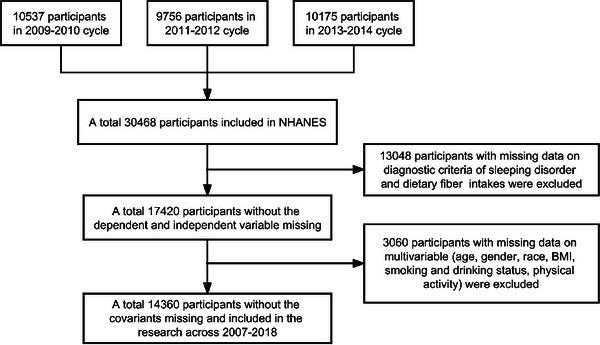
Flowchart of sample selection in NHANES 2009–2014.

Since 1999, NHANES has conducted a biennial survey on a nationally representative sample with a two‐year cycle, collecting data from approximately 5000 individuals each year (10,000 individuals per cycle). The survey employs a multi‐stage probability sampling design to obtain a representative sample. We selected data from three consecutive NHANES cycles conducted from 2009 to 2014 for our study, including 10,537 participants in 2009–2010, 9756 in 2011–2012, and 10,175 in 2013–2014. A total of 30,468 adult participants completed data collection. To ensure the completeness of information for each sample, we excluded 13,048 participants who lacked information on sleep disorders or DFI and 3060 participants who lacked information on covariates, including age, gender, race, BMI, smoking, alcohol consumption, and physical activity. Finally, our study included 14,360 eligible participants. The comprehensive process of participant selection is depicted in Figure 1.

### 2.2 DFI

The data on DFI in NHANES was obtained through a 24‐h dietary recall to assess dietary intake. Two 24‐h dietary recall interviews were conducted for all participants, with the first questionnaire administered at the Mobile Examination Center (MEC) during the initial examination. The second questionnaire was administered via telephone 3 to 10 days later, gathering information on all consumed foods. Subsequently, survey personnel utilized a specific coding system (the food coding system of the U.S. Department of Agriculture, USDA) to link each reported food item by participants to corresponding entries in the food composition database. The intake of each food item was multiplied by its fiber content in the food composition database, yielding the fiber intake contributed by each food item. The fiber intake for each food item was then aggregated, providing the overall DFI for participants during a specific time period. For data with no missing values in both interviews, the mean was calculated.

### 2.3 Sleep Disorders

The outcome variable consisted of participants diagnosed with sleep disorders by a physician. In the NHANES data personal interview questionnaire, individuals who responded “yes” to the question “Ever told by doctor have sleep disorder?” were defined as having sleep disorders. For further details, please refer to https://wwwn.cdc.gov/Nchs/Nhanes/2013‐2014/SLQ_H.htm.

### 2.4 Covariates

We collected factors related to DFI and sleep disorders as covariates, including demographic characteristics such as gender (male/female), race (Mexican American, Hispanic, non‐Hispanic white, non‐Hispanic black, and other), age (20–45, 45–69, > 69), BMI (< 25 kg/m^2^, 25–30 kg/m^2^, > 30 kg/m^2^), alcohol consumption, smoking status, physical activity, and energy intake. Alcohol consumption was defined as whether the participant had consumed 12 ounces of beer, 5 ounces of wine, or 1.5 ounces of liquor per day on average in the past year (Polen et al. 2010). Smoking status was classified as “Now smoking” for those who reported smoking “every day” or “some days” in response to the question “Do you now smoke?” and “Former smoking” for those who reported having smoked at least 100 cigarettes in their lifetime but were not current smokers; all others were classified as “Never smoking” (Wan et al. 2023). Physical activity (PA) data was derived from the NHANES Physical Activity Questionnaire (PAQ), and it was calculated based on the Metabolic Equivalent (MET) (Mendes et al. 2018), activity type, weekly frequency, and duration, using the following formula: PA (MET‐h/week) = MET × weekly frequency × duration of each physical activity (Chen et al. 2022, Ran et al. 2021). PA = 0 was defined as no physical activity, while the remaining participants were categorized into low and high physical activity groups based on PA. Finally, participants were grouped into the no physical activity group (< 1 MET‐h/week), low physical activity group (1–48 MET‐h/week), and high physical activity group (> 48 MET‐h/week). The energy intake (kcal/day) was calculated using the same method as the DFI (g/day) mentioned above, derived from the 24‐hour dietary recall data.

### 2.5 Statistical Analysis

Data analysis was manipulated utilizing R software (v4.2.1). “Tableone” package was utilized to generate baseline tables, and the “survey” package was used to analyze the survey data. Baseline characteristics of all participants were presented in tables, and separate tables were created for participants with and without sleep disorders. Categorical variables were reported as frequencies and percentages, while continuous variables were presented as means and standard deviations. Logistic regression was employed to construct models and investigate the association between DFI and sleep disorders. Stratified analyses were performed based on gender, age, race, BMI, smoking status, alcohol consumption, and physical activity, and the interaction between the stratification variables was tested utilizing the chi‐square test. Four models were constructed to mine the linkage between DFI and sleep disorders, adjusting for different confounding factors: a crude model with no adjustment, model I adjusted for age, gender, and race, model II adjusted for gender, age, race, BMI, smoking status, alcohol consumption, and physical activity, and model III adjusted for gender, age, race, BMI, smoking status, alcohol consumption, physical activity, and energy intake. Performing subgroup analysis of the above models using DFI as a continuous variable, stratified by gender and activity intensity factors. Odds ratios (ORs) and 95% confidence intervals (CIs) were utilized to exhibit the results, with statistical significance defined as a *p*‐value < 0.05.

## 3 Results

14,360 participants were included here. As illustrated in Table 1, no significant difference existed in the gender distribution, with 49.4% male and 50.6% female. A staggering 88.6% of participants fell within the age range of 20–69 years, constituting the majority, while non‐Hispanic whites emerged as the predominant racial group, making up 66.9% of the total. The distribution of BMI was relatively balanced. Participants were grouped in line with the presence or absence of sleep disorders, as shown in Table 2. Of the participants, 1301 (9.1%) reported having sleep disorders, with a slightly higher percentage of males (53.1%) than females (46.9%), but the difference was not statistically significant (*p* > 0.05). Significant differences existed in the distribution of age, race, BMI, physical activity, smoking status, and DFI between the sleep disorder and non‐sleep disorder groups.

**TABLE 1:** Overall characteristics distribution of participants.

Characteristics	*n* (%) / Mean ± SD
Overall	14360
Gender	
Female	7257 (50.6)
Male	7103 (49.4)
Age (years)	
20–45	6406 (48.1)
45–69	5689 (40.5)
> 69	2265 (11.4)
Race	
Mexican American	1998 (8.7)
Other Hispanic	1385 (5.6)
Non‐Hispanic White	6403 (66.9)
Non‐Hispanic Black	3035 (11.4)
Other race	1539 (7.4)
BMI (kg/m^2^)	
≤ 25	4252 (30.8)
25–30	4714 (33.1)
> 30	5394 (36.1)
Smoking	
Never smoking	7955 (56.0)
Former smoking	3437 (24.3)
Now Smoking	2968 (19.7)
Alcohol drinking	
No	3822 (21.5)
Yes	10538 (78.5)
PA	
No physical activity	3717 (22.0)
Low physical activity	6442 (47.1)
High physical activity	4201 (30.9)
Sleeping disorder	
Non‐sleeping disorder	13059 (90.5)
Sleeping disorder	1301 (9.5)
Energy (kcal)	2117.95 (843.48)
Dietary fiber (gm)	17.46 (9.38)

*Note*: *n* is unweighted; *n* (%), mean, and SD are adjusted for weights.

Abbreviations: BMI: body mass index; PA: physical activity.

**TABLE 2:** Characteristics distribution of participants in sleep disorder group.

Characteristics	Non‐sleep disorder	Sleep disorder	*p*‐value
Overall	13059	1301	
Gender			0.057
Female	6627 (50.9)	630 (46.9)	
Male	6432 (49.1)	671 (53.1)	
Age (years)			< 0.001*
20–45	6012 (49.7)	394 (32.9)	
45–69	5004 (39.0)	685 (55.0)	
> 69	2043 (11.3)	222 (12.1)	
Race			< 0.001*
Mexican American	1884 (9.1)	114 (5.3)	
Other Hispanic	1256 (5.6)	129 (5.1)	
Non‐Hispanic White	5708 (66.3)	695 (73.1)	
Non‐Hispanic Black	2759 (11.4)	276 (11.3)	
Other race	1452 (7.6)	87 (5.2)	
BMI (kg/m^2^)			< 0.001*
≤ 25	4059 (32.2)	193 (17.5)	
25–30	4393 (33.8)	321 (25.9)	
> 30	4607 (34.0)	787 (56.7)	
Smoking			<0.001*
Never smoking	7373 (57.2)	582 (44.6)	
Former smoking	3030 (23.5)	407 (31.4)	
Now Smoking	2656 (19.3)	312 (24.1)	
Alcohol drinking			1.00
No	3485 (21.5)	337 (21.5)	
Yes	9574 (78.5)	964 (78.5)	
PA			<0.001*
No physical activity	3259 (20.5)	458 (35.9)	
Low physical activity	5905 (47.8)	537 (40.2)	
High physical activity	3895 (31.6)	306 (23.8)	
Energy(kcal)	2123.61 (841.04)	2064.30 (864.75)	0.194
Dietary fiber (gm)	17.57 (9.47)	16.42 (8.37)	0.003*

*Note*: *n* is unweighted; *n* (%), mean, and SD are adjusted for weights.

Abbreviations: BMI: body mass index; PA: physical activity.

* indicates statistical significance (*p* < 0.05).

As shown in Table 3, in the weighted logistic regression model without adjusting for confounding factors, DFI was notably linked with a reduced risk of sleep disorders (OR: 0.99, 95% CI: 0.98–1.00, *p* = 0.005), while energy intake had no significant effect on the risk of sleep disorders (OR: 1.00, 95% CI: 1.00–1.00, *p* = 0.200).

**TABLE 3:** Logistic regression models based on dietary fiber and energy intake and sleep disorders.

Characteristic	OR	95% CI	*p*‐value
Dietary fiber (gm)	0.99	0.98–1.00	0.005*
Energy (kcal)	1.00	1.00–1.00	0.200

Abbreviation: CI, confidence interval.

* indicates statistical significance (*p* < 0.05).

A stratified analysis of the weighted logistic regression model for DFI and sleep disorders without adjusting for confounding variables was conducted, as shown in Table 4. Outcomes unmasked that, among females, DFI was tellingly linked with an improvement in sleep disorders (OR: 0.97, 95% CI: 0.96–0.98, *p* < 0.001), and among individuals with low physical activity levels, DFI was tellingly linked with an improvement in sleep disorders (OR: 0.98, 95% CI: 0.96–0.99, *p* < 0.001). In the model adjusted for all confounding factors, the interaction terms between gender (*p* = 0.017) and race (*c* = 0.014) with DFI exhibited significant effects. Conversely, factors such as physical activity, age, BMI, smoking status, alcohol consumption, and others did not show significant effects on the model (interaction terms *p* > 0.05).

**TABLE 4:** Multivariable stratified logistic regression models.

Participants	OR	95% CI	*p*‐value	*p* for interaction
Gender				0.017*
Female	0.97	0.96–0.98	<0.001*	
Male	0.99	0.98–1.00	0.120	
Age (years)				0.888
20–45	0.98	0.96–1.00	0.110	
45–69	0.99	0.97–1.00	0.043*	
> 69	0.98	0.95–1.01	0.200	
Race				0.014*
Mexican American	0.97	0.95–1.00	0.019*	
Other Hispanic	0.98	0.96–1.01	0.140	
Non‐Hispanic White	0.99	0.97–1.00	0.041*	
Non‐Hispanic Black	1.01	1.00–1.03	0.150	
Other race	0.97	0.94–0.99	0.009*	
BMI (kg/m^2^)				0.393
≤ 25	0.99	0.97–1.01	0.400	
25–30	0.98	0.96–1.00	0.049*	
> 30	1.00	0.98–1.01	0.400	
Smoking				0.504
Never smoking	0.99	0.97–1.00	0.100	
Former smoking	0.98	0.96–1.00	0.072	
Now smoking	1.00	0.98–1.02	> 0.900	
Alcohol drinking				0.211
No	0.98	0.96–1.00	0.020*	
Yes	0.99	0.98–1.00	0.025*	
PA				0.061
No physical activity	1.00	0.98–1.02	0.800	
Low physical activity	0.98	0.96–0.99	<0.001*	
High physical activity	1.00	0.99–1.02	> 0.900	

*Note*: Interaction term *p*‐values adjusted for gender, age, race, BMI, smoking, alcohol consumption, activity intensity.

Abbreviations: BMI: body mass index; PA: physical activity.

* indicates statistical significance (*p* < 0.05).

A weighted logistic regression model was constructed based on quartiles of DFI and different confounding factors, as shown in Table 5. In the crude model without adjustment for confounding factors, the Q4 group (OR: 0.76, 95% CI: 0.59–0.97, *p* = 0.026) showed significant results. In model I, which adjusted for population characteristics only, the Q3 (OR: 0.74, 95% CI: 0.56–0.98, *p* = 0.036) and Q4 (OR: 0.70, 95% CI: 0.55–0.90, *p* = 0.006) groups also exhibited notable associations. These results indicated increasing DFI as a significant protective factor against sleep disorders.

**TABLE 5:** Adjusted relationship models of different confounding factors stratified by quartiles of dietary fiber intake.

Models	Quantile	OR	95% CI	*p‐*value
Crude	Q1 (<11.10)	Ref.		
	Q2 (11.10–15.80)	0.96	0.80–1.16	0.700
	Q3 (15.80–21.95)	0.80	0.61–1.05	0.110
	Q4 (≥ 21.95)	0.76	0.59–0.97	0.026*
Model I	Q1 (<11.10)	Ref.		
	Q2 (11.10–15.80)	0.93	0.77–1.13	0.500
	Q3 (15.80–21.95)	0.74	0.56–0.98	0.036*
	Q4 (≥ 21.95)	0.70	0.55–0.90	0.006*
Model II	Q1 (<11.10)	Ref.		
	Q2 (11.10–15.80)	1.00	0.82–1.22	> 0.900
	Q3 (15.80–21.95)	0.88	0.67–1.14	0.300
	Q4 (≥ 21.95)	0.90	0.69–1.17	0.400
Model III	Q1 (<11.10)	Ref.		
	Q2 (11.10–15.80)	1.02	0.82–1.29	0.800
	Q3 (15.80–21.95)	0.92	0.70–1.20	0.500
	Q4 (≥ 21.95)	0.96	0.70–1.32	0.800

*Note*: Crude: Unadjusted; Model I: Adjusted for gender, age, and race; Model II: Adjusted for gender, age, race, BMI, smoking, alcohol consumption, and activity intensity; Model III: Adjusted for gender, age, race, BMI, smoking, alcohol consumption, activity intensity, and energy intake.

* indicates statistical significance (*p* < 0.05).

As shown in Table 6, a relationship model between DFI and sleep disorders was constructed after adjusting for different confounding factors, using DFI as a continuous variable. The crude group (OR: 0.99, 95% CI: 0.98–1.00, *p* = 0.005) and model I (OR: 0.98, 95% CI: 0.97–0.99, *p* = 0.002) were significant. Subgroup analysis was then performed for gender and physical activity level in the stratified model analysis. In the gender subgroup analysis, the crude model (OR: 0.97, 95% CI: 0.96–0.98, *p* < 0.001), model I (OR: 0.97, 95% CI: 0.95–0.98, *p* < 0.001), model II (OR: 0.98, 95% CI: 0.96–0.99, *p* = 0.004), and model III (OR: 0.98, 95% CI: 0.96–1.00, *p* = 0.021) in the female group all showed significant results, while no significant results were observed in the male sample. These suggested that mounting DFI was more beneficial for improving sleep disorders in female patients. In the subgroup analysis based on activity intensity, the results from all four models in the low physical activity group exhibited statistical significance (*p* < 0.05). However, no statistical significance was observed in the no physical activity group and high physical activity group (*p* > 0.05).

**TABLE 6:** Subgroup analysis of gender and activity intensity factors.

Participants	Models	OR	95% CI	*p*‐value
All participants	Crude	0.99	0.98–1.00	0.005*
	Model I	0.98	0.97–0.99	0.002*
	Model II	0.99	0.98–1.00	0.200
	Model III	1.00	0.98–1.01	0.600
Gender				
Female	Crude	0.97	0.96–0.98	<0.001*
	Model I	0.97	0.95–0.98	<0.001*
	Model II	0.98	0.96–0.99	0.004*
	Model III	0.98	0.96–1.00	0.021*
Male				
	Crude	0.99	0.98–1.00	0.120
	Model I	0.99	0.98–1.00	0.200
	Model II	1.00	0.99–1.01	0.800
	Model III	1.00	0.99–1.02	0.600
PA				
No physical activity	Crude	1.00	0.98–1.02	0.800
	Model I	0.99	0.97–1.01	0.300
	Model II	0.99	0.97–1.01	0.400
	Model III	1.01	0.98–1.04	0.600
Low physical activity	Crude	0.98	0.96–0.99	<0.001*
	Model I	0.97	0.96–0.99	<0.001*
	Model II	0.98	0.97–1.00	0.022*
	Model III	0.98	0.96–1.00	0.026*
High physical activity	Crude	1.00	0.99–1.02	> 0.900
	Model I	1.00	0.99–1.02	0.900
	Model II	1.01	0.99–1.02	0.300
	Model III	1.01	0.99–1.02	0.300

Abbreviation: PA, physical activity.

* indicates statistical significance (*p* < 0.05).

In the female subgroup, an increase in DFI was observed to be more favorable for improving sleep disorders. Consequently, we further grouped females based on menopausal status (non‐postmenopausal vs. postmenopausal). The analysis revealed differences in BMI and sleep disorders (*p* < 0.05), but no distinction in DFI (Table S1). By exploring the Logistic regression model for DFI and sleep disorders in women based on grouping, it was found that an increased intake of dietary fiber in postmenopausal women significantly improved sleep disorders, especially in participants with BMI > 30 kg/m^2^ (Tables S2 and S3).

## 4 Discussion

This study found a significant correlation between DFI and sleep disorders, with an inverse association between increased DFI and reduced risk of sleep disorders. This phenomenon was more pronounced in women, particularly those in the menopausal stage and women with a BMI > 30 kg/m^2^. Moreover, the effect of DFI on improving sleep disorders may also be related to the intensity of physical activity. The improvement was more evident in individuals with low activity levels, while no such effect was observed in those engaging in high‐intensity activities.

The molecular mechanism underlying the improvement of sleep disorders with increased DFI may originate from the regulation of the gut microbiota by dietary fiber. Research by Awika, Rose, and Simsek (2018) has reported a negative correlation between total DFI and inflammation. Inflammation plays a crucial role in human sleep and is positively correlated with sleep disorders (Mills et al. 2007). Dietary fiber can regulate the gut environment and alter the gut microbiota. Gut microbes convert fiber to short‐chain fatty acids, which have been suggested to reduce intestinal permeability and uptake of gut microbiota‐derived bacterial antigens, including lipopolysaccharide, an endotoxin known to instigate an inflammatory response (Kantor et al. 2013, Fougerat et al. 2020). In 2022, Tang et al. (2022) reported that dietary fiber metabolism in the colon increases the type and quantity of beneficial bacteria and their representative short‐chain fatty acids in the gut, improves damaged intestinal barriers, stimulates the secretion of sleep cytokines through the “gut‐brain axis,” and inhibits inflammatory pathways, and increases serotonin secretion to improve sleep disorders. A randomized crossover inpatient study, by examining the relationship between food intake and nocturnal sleep, also discovered that an increase in DFI is associated with deeper sleep (St‐Onge et al. 2016). A high‐fiber diet can alter the pH and permeability of the gut, reducing inflammation and consequently lowering the risk of sleep disorders. The regulatory mechanism of dietary fiber on sleep disorders may involve the gut microbiota stimulating the secretion of sleep‐related cytokines in the brain regions through the “gut‐brain axis,” thus alleviating sleep disorders.

As widely known, insomnia is the most common type of sleep disorder, exhibiting significant gender differences. The risk of insomnia in females is 1.6 times that in males, and the prevalence of insomnia increases with age (Li et al. 2002; Zhang and Wing 2006; Brito et al. 2021). There exists a crucial biological difference between the sexes, attributed to variations in sex hormone levels. Female sex hormones may impact emotional symptoms, as evident in women with premenstrual syndrome (PMS), where 48% of females experience PMS symptoms (Direkvand‐Moghadam et al. 2014), reporting mood swings and fatigue in the week preceding menstruation (Baker and Driver 2007; Halbreich et al. 2003). Fatigue is also a symptom of premenstrual dysphoric disorder (PMDD), suggesting a potential influence on sleep quality. During the transitional phase of menopause, when sex hormones fluctuate significantly and eventually decrease, sleep problems, reported as “sleep difficulties” and “sleep disorders,” become a common issue (Kravitz et al. 2003). Our results indicated that an increased DFI in postmenopausal women could significantly improve sleep disorders, particularly in participants with BMI > 30 kg/m^2^. This could be attributed to the likelihood of elevated blood glucose levels in individuals with a high BMI index. A prospective cohort study targeting postmenopausal women also found that a high dietary glycemic index (GI) is a risk factor for insomnia in postmenopausal women, where the dietary source of sugars influences GI, and a higher fiber content in food slows carbohydrate metabolism and reduces GI, thereby lowering the incidence rate (Gangwisch et al. 2020; Jenkins and Jenkins 1985). Changing dietary habits and incorporating fiber‐rich carbohydrates as alternatives to high‐GI foods could serve as a potential therapeutic approach and primary preventive measure for insomnia in postmenopausal women. Another significant reason for the prominent reduction in the risk of sleep disorders with increased DFI in the female population may be the gender differences in the physiological regulation of neurotransmitters, such as serotonin. In females, there is a stronger binding capacity between serotonin and its receptors compared to males (Hilz and Gore 2022). This ultimately manifests as a more pronounced improvement in sleep disorders with increased DFI. However, the specific mechanisms require further validation and investigation.

This study found that the improvement in sleep disorders due to DFI was more pronounced in individuals engaging in low‐intensity physical activity, while no such effect was observed in those with high‐intensity physical activity. This finding suggests a potential synergistic effect between DFI and physical activity, with the benefits of dietary fiber being more apparent in the context of low‐intensity physical activity. Several biological mechanisms may be involved. First, different types of dietary fiber or their fermentation products, such as short‐chain fatty acids (SCFAs), are known to have beneficial effects on the body (Cronin et al. 2021). At the same time, there is evidence that physical exercise can increase microbial metabolites like SCFAs (Hughes 2019), and changes in SCFA levels have been linked to sleep quality, with lower SCFA levels associated with shorter sleep duration (Shimizu et al. 2023). Additionally, dietary fiber has been linked to reducing chronic inflammation and oxidative stress, as well as improving dietary inflammation index scores, all of which are related to sleep quality (Frith et al. 2018; Coxon, Nishihira, and Hepsomali 2024; Qamar et al. 2022; Atrooz and Salim 2020). Therefore, the relationship between low‐intensity exercise and sleep may be explained by the modulation of inflammation levels in the body through dietary fiber intake. Physical activity can regulate sleep by affecting serotonin levels in the brain (Melancon, Lorrain, and Dionne 2014), with the serotoninergic system's increased activity modulating the sleep‐wake cycle. It is worth noting that some of the studies supporting this conclusion come from animal models. For example, Park et al. (2022) found in a mouse experiment that the levels of fatty acids such as palmitic acid and oleamide significantly increased after exercise, mainly due to increased serotonin activity during exercise. These studies suggest that by increasing physical activity, serotonin synthesis and release can be enhanced, which may also improve sleep. However, the exact manifestation of these mechanisms in humans requires further validation. Overall, regular low‐intensity exercise may improve sleep quality through the regulation of inflammation and neurotransmitters. In contrast, excessive physical activity in high‐intensity exercise may increase stress levels in the body, negatively impacting sleep quality. Given the positive effects of regular physical activity, it is recommended to incorporate fiber‐rich foods into daily life, combined with moderate and regular exercise, to improve sleep issues.

This study holds significance in advocating for the general public to reduce the risk of sleep disorders through a healthy diet by increasing DFI. However, it is important to acknowledge certain limitations. Firstly, due to the cross‐sectional design of NHANES, we cannot establish a causal relationship between DFI and sleep disorders. Secondly, the subjective self‐reporting used to assess sleep disorders in the NHANES design may introduce individual variations, and the results might be less accurate compared to objective measurements. Although the large population sample and the complex multi‐stage sampling design partly compensate for this limitation, there remains a possibility of inaccuracy. Additionally, our analysis is based on NHANES data collected from the U.S. population, introducing potential regional biases. Lastly, residual confounding from unmeasured factors, such as medical conditions, dietary patterns, occupational status, and more, could introduce bias to our results. Future research, more in‐depth study designs, and long‐term tracking are needed to further validate the role of dietary fiber in sleep health and provide more specific guidance for related prevention and treatment.

## Author Contributions

**Yijun Chen**: conceptualization, funding acquisition, writing–original draft, formal analysis, project administration. **Zengchen Zhao**: conceptualization, methodology, software, data curation, writing–original draft. **Weijun Ding**: investigation, validation, visualization, project administration, writing–review and editing. **Zhenting Zhou**: software, data curation, supervision, resources, project administration, writing–review and editing. **Meihong Xiao**: conceptualization, supervision, methodology, funding acquisition, writing–review and editing, validation.

## Ethics Statement

The authors have nothing to report.

## Conflicts of Interest

The authors declare no conflicts of interest.

### Peer Review

The peer review history for this article is available at https://publons.com/publon/10.1002/brb3.70123.

## Supporting information



Supporting Materials.

## Data Availability

The data in this study are available from the corresponding author on reasonable request.

## References

[brb370123-bib-0001] Al Lihabi, A. 2023. “A Literature Review of Sleep Problems and Neurodevelopment Disorders.” Front Psychiatry 14: 1122344. 10.3389/fpsyt.2023.1122344. 36911135 PMC9995546

[brb370123-bib-0002] Antza, C. , G. Kostopoulos , S. Mostafa , K. Nirantharakumar , and A. Tahrani . 2021. “The Links Between Sleep Duration, Obesity and Type 2 Diabetes Mellitus.” Journal of Endocrinology 252, no. 2: 125–141. 10.1530/JOE-21-0155. 34779405 PMC8679843

[brb370123-bib-0003] Atrooz, F. , and S. Salim . 2020. “Sleep Deprivation, Oxidative Stress and Inflammation.” Advances in Protein Chemistry and Structural Biology 119: 309–336. 10.1016/bs.apcsb.2019.03.001. 31997771

[brb370123-bib-0004] Awika, J. M. , D. J. Rose , and S. Simsek . 2018. “Complementary Effects of Cereal and Pulse Polyphenols and Dietary fiber on Chronic Inflammation and Gut Health.” Food & Function 9, no. 3: 1389–1409. 10.1039/c7fo02011b. 29532826

[brb370123-bib-0005] Baker, F. C. , and H. S. Driver . 2007. “Circadian Rhythms, Sleep, and the Menstrual Cycle.” Sleep Medicine 8, no. 6: 613–622. 10.1016/j.sleep.2006.09.011. 17383933

[brb370123-bib-0006] Basu, A. , D. Feng , P. Planinic , J. L. Ebersole , T. J. Lyons , and J. M. Alexander . 2021. “Dietary Blueberry and Soluble Fiber Supplementation Reduces Risk of Gestational Diabetes in Women With Obesity in a Randomized Controlled Trial.” Journal of Nutrition 151, no. 5: 1128–1138. 10.1093/jn/nxaa435. 33693835 PMC8112774

[brb370123-bib-0007] Brito, R. S. , C. Dias , A. Afonso Filho , and C. Salles . 2021. “Prevalence of Insomnia in Shift Workers: A Systematic Review.” Sleep Science 14, no. 1: 47–54. 10.5935/1984-0063.20190150. 34104337 PMC8157778

[brb370123-bib-0008] Buttner‐Teleaga, A. , Y. T. Kim , T. Osel , and K. Richter . 2021. “Sleep Disorders in Cancer—A Systematic Review.” International Journal of Environmental Research and Public Health 18, no. 21: 11696. 10.3390/ijerph182111696. 34770209 PMC8583058

[brb370123-bib-0009] Chellappa, S. L. , and D. Aeschbach . 2022. “Sleep and Anxiety: From Mechanisms to Interventions.” Sleep Medicine Reviews 61: 101583. 10.1016/j.smrv.2021.101583. 34979437

[brb370123-bib-0010] Chen, L. , M. Cai , H. Li , et al. 2022. “Risk/Benefit Tradeoff of Habitual Physical Activity and Air Pollution on Chronic Pulmonary Obstructive Disease: Findings From a Large Prospective Cohort Study.” BMC Medicine 20, no. 1: 70. 10.1186/s12916-022-02274-8. 35220974 PMC8883705

[brb370123-bib-0011] Coxon, C. , J. Nishihira , and P. Hepsomali . 2024. “Dietary Inflammatory Index, Sleep Duration, and Sleep Quality: A Systematic Review.” Nutrients 16, no. 6: 890. 10.3390/nu16060890. 38542801 PMC10974932

[brb370123-bib-0012] Cronin, P. , S. A. Joyce , P. W. O'Toole , and E. M. O'Connor . 2021. “Dietary Fibre Modulates the Gut Microbiota.” Nutrients 13, no. 5: 1655. 10.3390/nu13051655. 34068353 PMC8153313

[brb370123-bib-0013] Direkvand‐Moghadam, A. , K. Sayehmiri , A. Delpisheh , and S. Kaikhavandi . 2014. “Epidemiology of Premenstrual Syndrome (PMS)—A Systematic Review and Meta‐Analysis Study.” Journal of Clinical and Diagnostic Research 8, no. 2: 106–109. 10.7860/JCDR/2014/8024.4021. 24701496 PMC3972521

[brb370123-bib-0014] Fougerat, A. , A. Montagner , N. Loiseau , H. Guillou , and W. Wahli . 2020. “Peroxisome Proliferator‐Activated Receptors and Their Novel Ligands as Candidates for the Treatment of Non‐Alcoholic Fatty Liver Disease.” Cells 9, no. 7: 1638. 10.3390/cells9071638. 32650421 PMC7408116

[brb370123-bib-0015] Frith, E. , N. Shivappa , J. R. Mann , J. R. Hebert , M. D. Wirth , and P. D. Loprinzi . 2018. “Dietary Inflammatory Index and Memory Function: Population‐Based National Sample of Elderly Americans.” British Journal of Nutrition 119, no. 5: 552–558. 10.1017/S0007114517003804. 29361990 PMC5839966

[brb370123-bib-0016] Gangwisch, J. E. , L. Hale , M. P. St‐Onge , et al. 2020. “High Glycemic Index and Glycemic Load Diets as Risk Factors for Insomnia: Analyses From the Women's Health Initiative.” American Journal of Clinical Nutrition 111, no. 2: 429–439. 10.1093/ajcn/nqz275. 31828298 PMC6997082

[brb370123-bib-0017] Guan, Z. W. , E. Z. Yu , and Q. Feng . 2021. “Soluble Dietary Fiber, One of the Most Important Nutrients for the Gut Microbiota.” Molecules 26, no. 22: 6802. 10.3390/molecules26226802. 34833893 PMC8624670

[brb370123-bib-0018] Halbreich, U. , J. Borenstein , T. Pearlstein , and L. S. Kahn . 2003. “The Prevalence, Impairment, Impact, and Burden of Premenstrual Dysphoric Disorder (PMS/PMDD).” Psychoneuroendocrinology 28, no. S3: 1–23. 10.1016/s0306-4530(03)00098-2. 12892987

[brb370123-bib-0019] Han, B. , W. Z. Chen , Y. C. Li , J. Chen , and Z. Q. Zeng . 2020. “Sleep and Hypertension.” Sleep & Breathing = Schlaf & Atmung 24, no. 1: 351–356. 10.1007/s11325-019-01907-2. 31402441 PMC7127991

[brb370123-bib-0020] Hepsomali, P. , and J. A Groeger . 2021. “Diet, Sleep, and Mental Health: Insights From the UK Biobank Study.” Nutrients 13, no. 8: 2573. 10.3390/nu13082573. 34444731 PMC8398967

[brb370123-bib-0021] Hilz, E. N. , and A. C. Gore . 2022. “Sex‐Specific Effects of Endocrine‐Disrupting Chemicals on Brain Monoamines and Cognitive Behavior.” Endocrinology 163, no. 10: bqac128. 10.1210/endocr/bqac128. 35939362 PMC9419695

[brb370123-bib-0022] Hughes, R. L. 2019. “A Review of the Role of the Gut Microbiome in Personalized Sports Nutrition.” Frontiers in Nutrition 6: 191. 10.3389/fnut.2019.00191. 31998739 PMC6966970

[brb370123-bib-0023] Jenkins, D. J. , and A. L. Jenkins . 1985. “Dietary Fiber and the Glycemic Response.” Proceedings of the Society for Experimental Biology and Medicine 180, no. 3: 422–431. 10.3181/00379727-180-42199. 3001740

[brb370123-bib-0024] Kantor, E. D. , J. W. Lampe , M. Kratz , and E. White . 2013. “Lifestyle Factors and Inflammation: Associations by Body Mass Index.” PLoS ONE 8, no. 7: e67833. 10.1371/journal.pone.0067833. 23844105 PMC3699492

[brb370123-bib-0025] Kravitz, H. M. , P. A. Ganz , J. Bromberger , L. H. Powell , K. Sutton‐Tyrrell , and P. M. Meyer . 2003. “Sleep Difficulty in Women at Midlife: A Community Survey of Sleep and the Menopausal Transition.” Menopause 10, no. 1: 19–28. 10.1097/00042192-200310010-00005. 12544673

[brb370123-bib-0026] Li, R. H. , Y. K. Wing , S. C. Ho , and S. Y. Fong . 2002. “Gender Differences in Insomnia—A Study in the Hong Kong Chinese Population.” Journal of Psychosomatic Research 53, no. 1: 601–609. 10.1016/s0022-3999(02)00437-3. 12127178

[brb370123-bib-0027] McDermott, M. , D. L. Brown , and R. D. Chervin . 2018. “Sleep Disorders and the Risk of Stroke.” Expert Review of Neurotherapeutics 18, no. 7: 523–531. 10.1080/14737175.2018.1489239. 29902391 PMC6300163

[brb370123-bib-0028] Melancon, M. O. , D. Lorrain , and I. J. Dionne . 2014. “Exercise and Sleep in Aging: Emphasis on Serotonin.” Pathologie Biologie 62, no. 5: 276–283. 10.1016/j.patbio.2014.07.004. 25104243

[brb370123-bib-0029] Mendes, M. A. , I. da Silva , V. Ramires , et al. 2018. “Metabolic Equivalent of Task (METs) Thresholds as an Indicator of Physical Activity Intensity.” PLoS ONE 13, no. 7: e0200701. 10.1371/journal.pone.0200701. 30024953 PMC6053180

[brb370123-bib-0030] Meng, X. , Y. Li , S. Li , et al. 2017. “Dietary Sources and Bioactivities of Melatonin.” Nutrients 9, no. 4: 367. 10.3390/nu9040367. 28387721 PMC5409706

[brb370123-bib-0031] Mills, P. J. , R. von Kanel , D. Norman , L. Natarajan , M. G. Ziegler , and J. E. Dimsdale . 2007. “Inflammation and Sleep in Healthy Individuals.” Sleep 30, no. 6: 729–735. 10.1093/sleep/30.6.729. 17580594 PMC1978353

[brb370123-bib-0032] Offringa, L. C. , J. C. Hartle , J. Rigdon , and C. D. Gardner . 2021. “Changes in Quantity and Sources of Dietary Fiber From Adopting Healthy Low‐Fat vs. Healthy Low‐Carb Weight Loss Diets: Secondary Analysis of DIETFITS Weight Loss Diet Study.” Nutrients 13, no. 10: 3625. 10.3390/nu13103625. 34684626 PMC8539701

[brb370123-bib-0033] Park, J. S. , Y. J. Kim , W. Heo , and S. Kim . 2022. “The Study of Variation of Metabolites by Sleep Deficiency, and Intervention Possibility of Aerobic Exercise.” International Journal of Environmental Research and Public Health 19, no. 5: 2774. 10.3390/ijerph19052774. 35270465 PMC8910362

[brb370123-bib-0034] Pavlova, M. K. , and V. Latreille . 2019. “Sleep Disorders.” American Journal of Medicine 132, no. 3: 292–299. 10.1016/j.amjmed.2018.09.021. 30292731

[brb370123-bib-0035] Perez, M. N. , and R. M. E Salas . 2020. “Insomnia.” Continuum 26, no. 4: 1003–1015. 10.1212/CON.0000000000000879. 32756233

[brb370123-bib-0036] Polen, M. R. , C. A. Green , N. A. Perrin , B. M. Anderson , and C. M. Weisner . 2010. “Drinking Patterns, Gender and Health I: Attitudes and Health Practices.” Addiction Research & Theory 18, no. 2: 122–142. 10.3109/16066350903398486. 23946720 PMC3740444

[brb370123-bib-0037] Qamar, A. , Z. Haque , S. Zehra , and M. S. Baig . 2022. “Obstructive Sleep Apnoea: Potential Role of Tumour Necrosis Factor Alpha as a Circulating Biomarker.” Journal of the Pakistan Medical Association 72, no. 7: 1350–1354. 10.47391/JPMA.3239. 36156559

[brb370123-bib-0038] Ran, J. , Y. Zhang , L. Han , et al. 2021. “The Joint Association of Physical Activity and Fine Particulate Matter Exposure With Incident Dementia in Elderly Hong Kong Residents.” Environment International 156: 106645. 10.1016/j.envint.2021.106645. 34015665

[brb370123-bib-0039] Schoeneck, M. , and D. Iggman . 2021. “The Effects of Foods on LDL Cholesterol Levels: A Systematic Review of the Accumulated Evidence From Systematic Reviews and Meta‐Analyses of Randomized Controlled Trials.” Nutrition, Metabolism and Cardiovascular Diseases 31, no. 5: 1325–1338. 10.1016/j.numecd.2020.12.032. 33762150

[brb370123-bib-0040] Shimizu, Y. , R. Yamamura , Y. Yokoi , et al. 2023. “Shorter Sleep Time Relates to Lower Human Defensin 5 Secretion and Compositional Disturbance of the Intestinal Microbiota Accompanied by Decreased Short‐Chain Fatty Acid Production.” Gut Microbes 15, no. 1: 2190306. 10.1080/19490976.2023.2190306. 36945116 PMC10038026

[brb370123-bib-0041] Simon, S. , H. Rahat , A. M. Carreau , et al. 2020. “Poor Sleep Is Related to Metabolic Syndrome Severity in Adolescents with PCOS and Obesity.” Journal of Clinical Endocrinology and Metabolism 105, no. 4: e1827–1834. 10.1210/clinem/dgz285. 31901092 PMC7059992

[brb370123-bib-0042] Song, M. , A. T. Chan , and J. Sun . 2020. “Influence of the Gut Microbiome, Diet, and Environment on Risk of Colorectal Cancer.” Gastroenterology 158, no. 2: 322–340. 10.1053/j.gastro.2019.06.048. 31586566 PMC6957737

[brb370123-bib-0043] St‐Onge, M. P. , A. Roberts , A. Shechter , and A. R. Choudhury . 2016. “Fiber and Saturated Fat Are Associated With Sleep Arousals and Slow Wave Sleep.” Journal of Clinical Sleep Medicine 12, no. 1: 19–24. 10.5664/jcsm.5384. 26156950 PMC4702189

[brb370123-bib-0044] Swann, O. G. , M. Kilpatrick , M. Breslin , and W. H. Oddy . 2020. “Dietary Fiber and Its Associations With Depression and Inflammation.” Nutrition Reviews 78, no. 5: 394–411. 10.1093/nutrit/nuz072. 31750916

[brb370123-bib-0045] Tang, M. , X. Song , W. Zhong , Y. Xie , Y. Liu , and X. Zhang . 2022. “Dietary Fiber Ameliorates Sleep Disturbance Connected to the Gut‐Brain Axis.” Food & Function 13, no. 23: 12011–12020. 10.1039/d2fo01178f. 36373848

[brb370123-bib-0046] Vernia, F. , S. Longo , G. Stefanelli , A. Viscido , and G. Latella . 2021. “Dietary Factors Modulating Colorectal Carcinogenesis.” Nutrients 13, no. 1: 143. 10.3390/nu13010143. 33401525 PMC7824178

[brb370123-bib-0047] Wan, B. , P. Lin , M. Wang , et al. 2023. “The Association Between Dietary Inflammatory Index and Cognitive Function in Adults With/Without Chronic Kidney Disease.” Frontiers in Nutrition 10: 1279721. 10.3389/fnut.2023.1279721. 38075216 PMC10703050

[brb370123-bib-0048] Zhang, B. , and Y. K. Wing . 2006. “Sex Differences in Insomnia: A Meta‐Analysis.” Sleep 29, no. 1: 85–93. 10.1093/sleep/29.1.85. 16453985

[brb370123-bib-0049] Zhang, S. , S. X. Liu , Q. J. Wu , et al. 2023. “Association of Dietary Fiber With Subjective Sleep Quality in Hemodialysis Patients: A Cross‐Sectional Study in China.” Annals of Medicine 55, no. 1: 558–571. 10.1080/07853890.2023.2176541. 36752281 PMC9930787

