# Supplementary material for: Association Between Dietary Fiber Intake and Sleep Disorders: Based on the NHANES Database
Source: Brain Behav. 2024 Nov 5;14(11):e70123. doi: 10.1002/brb3.70123 (PMC11538121; doi:10.1002/brb3.70123)
Supplement: Supplementary file 1 — Supporting Materials. [file BRB3-14-e70123-s001.docx]

**Supplementary Table 1. Distribution characteristics of female participants**

| **Characteristics** | **Non-postmenopausal** | **Postmenopausal** | **P Value** |
| --- | --- | --- | --- |
| **Overall** | 4869 (69.6) | 2388 (30.4) |  |
| BMI (kg/m2) |  |  | <0.001* |
| <=25 | 1643 (37.2) | 585 (27.7) |  |
| 25-30 | 1297 (26.7) | 742 (31.6) |  |
| >30 | 1929 (36.2) | 1061 (40.8) |  |
| **Sleeping disorder** |  |  | 0.030* |
| No | 4479 (92.1) | 2148 (89.1) |  |
| Yes | 390 (7.9) | 240 (10.9) |  |
| **Dietary fiber (gm)** | 15.75 (8.03) | 15.80 (7.53) | 0.888 |
| Postmenopausal status was defined based on a self-reported reproductive health questionnaire. Women who answered “No” to the question “Have you had at least one menstrual period in the last 12 months?” were considered postmenopausal, followed by the answer “hysterectomy” or “menopause/life changes” to the question “What was the reason for not having a period in the past 12 months?”. BMI: Body Mass Index.  * indicates statistical significance (p < 0.05). | | | |

**Supplementary Table 2. Logistic regression model based on dietary fiber and sleep disorders in female**

| **Female** | **N (%)** | OR | 95% CI | P-value |
| --- | --- | --- | --- | --- |
| **Postmenopausal** |  |  |  |  |
| No | 4869 (69.6) | 0.99 | 0.96-1.01 | 0.200 |
| Yes | 2388 (30.4) | 0.97 | 0.95-0.99 | 0.006 * |
| Adjusted for age, race, BMI, smoking, alcohol consumption, activity intensity, energy intake. Note: CI represents confidence interval.  * indicates statistical significance (p < 0.05). | | | | |

**Supplementary Table 3. Logistic regression model of dietary fiber and sleep disorders based on different BMI in postmenopausal female**

| **Postmenopausal** | **N (%)** | OR | 95% CI | P-value |
| --- | --- | --- | --- | --- |
| BMI (kg/m2) |  |  |  |  |
| <=25 | 585 (27.7) | 0.99 | 0.89-1.09 | 0.800 |
| 25-30 | 742 (31.6) | 0.99 | 0.94-1.03 | 0.500 |
| >30 | 1061 (40.8) | 0.95 | 0.92-0.99 | 0.011 * |
| Adjusted for age, race, smoking, alcohol consumption, activity intensity, energy intake.  Note: CI represents confidence interval, * denotes P<0.05. BMI: Body Mass Index.  * indicates statistical significance (p < 0.05). | | | | |
